# Association of physicians’ Big Five personality traits with shared decision-making in patients with SLE

**DOI:** 10.1093/rheumatology/keaf288

**Published:** 2025-05-26

**Authors:** Shigemi Morishita, Ken-ei Sada, Masataka Kudo, Naofumi Dobashi, Sho Sasaki, Ryusuke Yoshimi, Natsuki Sakurai, Chiharu Hidekawa, Yasuhiro Shimojima, Dai Kishida, Takanori Ichikawa, Yoshia Miyawaki, Keigo Hayashi, Kenta Shidahara, Yuichi Ishikawa, Nao Oguro, Nobuyuki Yajima, Noriaki Kurita, Narufumi Suganuma

**Affiliations:** Department of Surgery, Oida Hospital, Sukumo, Japan; Department of Clinical Epidemiology, Kochi Medical School, Nankoku, Japan; Department of Clinical Epidemiology, Kochi Medical School, Nankoku, Japan; Department of Clinical Epidemiology, Kochi Medical School, Nankoku, Japan; Department of General Internal Medicine, Iizuka Hospital, Fukuoka, Japan; Department of Clinical Epidemiology, Kochi Medical School, Nankoku, Japan; Department of Internal Medicine, Kochi Prefectural Hata-Kenmin Hospital, Sukumo, Japan; Section of Education for Clinical Research, Kyoto University Hospital, Kyoto, Japan; Center for Innovative Research for Communities and Clinical Excellence (CiRC2LE), Fukushima Medical University, Fukushima, Japan; Department of Stem Cell and Immune Regulation, Yokohama City University Graduate School of Medicine, Yokohama, Japan; Clinical Laboratory Department, Yokohama City University Hospital, Yokohama, Japan; Department of Stem Cell and Immune Regulation, Yokohama City University Graduate School of Medicine, Yokohama, Japan; Department of Stem Cell and Immune Regulation, Yokohama City University Graduate School of Medicine, Yokohama, Japan; Department of Medicine (Neurology and Rheumatology), Shinshu University School of Medicine, Matsumoto, Japan; Department of Medicine (Neurology and Rheumatology), Shinshu University School of Medicine, Matsumoto, Japan; Department of Medicine (Neurology and Rheumatology), Shinshu University School of Medicine, Matsumoto, Japan; Department of Nephrology, Rheumatology, Endocrinology and Metabolism, Okayama University Graduate School of Medicine, Dentistry and Pharmaceutical Science, Okayama, Japan; Department of Nephrology, Rheumatology, Endocrinology and Metabolism, Okayama University Graduate School of Medicine, Dentistry and Pharmaceutical Science, Okayama, Japan; Department of Nephrology, Rheumatology, Endocrinology and Metabolism, Okayama University Graduate School of Medicine, Dentistry and Pharmaceutical Science, Okayama, Japan; The First Department of Internal Medicine, School of Medicine, University of Occupational and Environmental Health, Kitakyushu, Japan; Division of Rheumatology, Department of Medicine, Showa University of Medicine, Tokyo, Japan; Department of Clinical Epidemiology, Graduate School of Medicine, Fukushima Medical University, Fukushima, Japan; Division of Rheumatology, Department of Medicine, Showa University of Medicine, Tokyo, Japan; Center for Innovative Research for Communities and Clinical Excellence, Fukushima Medical University, Fukushima, Japan; Department of Healthcare Epidemiology, School of Public Health in Graduate School of Medicine, Kyoto University, Kyoto, Japan; Division of Rheumatology, Department of Medicine, Showa University of Medicine, Tokyo, Japan; Department of Clinical Epidemiology, Graduate School of Medicine, Fukushima Medical University, Fukushima, Japan; Department of Innovative Research and Education for Clinicians and Trainees (DiRECT), Fukushima Medical University Hospital, Fukushima, Japan; Department of Environment Medicine, Kochi Medical School, Nankoku, Japan; MEDi Center, Kochi University, Kochi, Japan

**Keywords:** SLE, shared decision-making, Big Five personality traits, cluster analysis

## Abstract

**Objectives:**

Recent European League Against Rheumatism guidelines highlight the importance of shared decision-making (SDM) in SLE treatment. This study investigated the relationship between physicians’ personality traits and patient-rated SDM.

**Methods:**

Using baseline data from the Trust Measurements for Physicians and Patients with SLE (TRUM2-SLE) study, physicians’ personality traits were assessed by the Japanese version of the 10-Item Personality Inventory (TIPI-J) scale (scores of 1–7 points each), while SDM was evaluated using the patient-reported nine-item SDM Questionnaire (SDM-Q-9) scale (total 0–100 points). Linear mixed-effects models with cluster-robust variance estimation analysed the association between TIPI-J scores and SDM-Q-9 scores. A cluster analysis was also performed to group physicians based on personality patterns, and the association between these clusters and SDM-Q-9 was evaluated.

**Results:**

Among 493 patients, the median SDM-Q-9 score was 75.6. Among 43 physicians, the median TIPI-J scores were as follows: extraversion, 4.0; agreeableness, 5.0; conscientiousness, 3.0; neuroticism, 3.5 and openness, 4.0. Conscientiousness (*β* = −1.67; 95% CI, −3.02 to −0.33) and neuroticism (*β* = −2.06; 95% CI −4.08 to −0.04) correlated negatively with SDM-Q-9 scores. A clustering analysis identified three physician groups; those in Cluster 2, characterized by low conscientiousness and neuroticism and labelled ‘lower perfectionism’, had significantly higher SDM-Q-9 scores than those in Clusters 1 and 3.

**Conclusion:**

A physician’s perfectionist tendencies may be a barrier to the SDM process with patients.

## Graphical abstract

**Figure:**
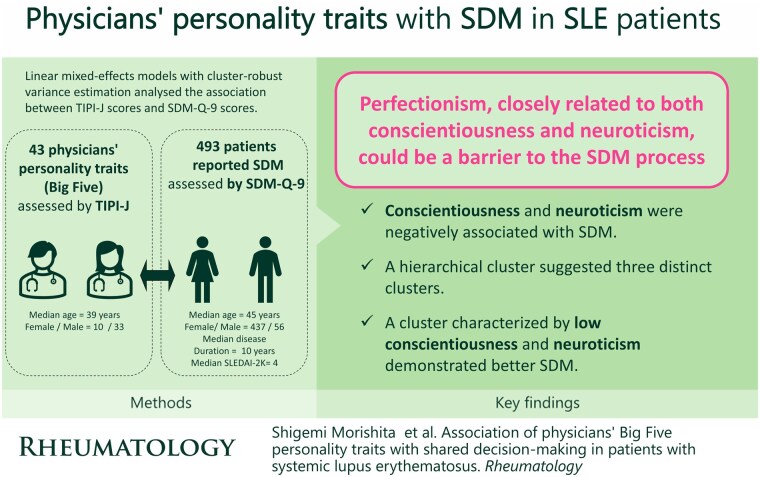

Rheumatology key messages

- Physicians' conscientiousness and neuroticism negatively affect shared decision-making (SDM).
- One physician cluster with low conscientiousness and neuroticism was characterized as having ‘low perfectionism’.
- Physicians' perfectionism could be a barrier to the SDM process.

## Introduction

SLE is a complex autoimmune disease. Despite recent advances in drug treatment strategies, effectively managing SLE remains challenging. Patients with SLE may experience a wide range of symptoms and organ involvement, both from the primary disease itself and from the medications used to treat it. As a result, personalized treatment approaches are needed to address these ongoing SLE management challenges [1]. The latest recommendations from the European League Against Rheumatism emphasize the importance of shared decision-making (SDM) in the personalized and intensive management of SLE [2].

SDM is a collaborative approach in which physicians and patients work together to make healthcare decisions by combining the best available evidence and patient values and preferences [3–5]. SDM has become increasingly important in modern healthcare, especially in managing chronic conditions such as SLE [6]. High-quality SDM is expected to play a key role in achieving treatment goals for patients with SLE, who may require ongoing treatment adjustments [7].

Physicians’ personality traits meaningfully impact patient experience, engagement and treatment efficacy, while clinical competence remains paramount [8]. The Big Five personality traits, also known as the Five-Factor Model, include extraversion, agreeableness, conscientiousness, neuroticism and openness and are widely used in personality research [9, 10]. Previous reports showed that high openness and agreeableness levels are linked to greater in-patient satisfaction [11]. Physicians with higher empathy and agreeableness levels tend to communicate better with in-patients, and these traits are associated with in-patient decision-making [12, 13]. Previous studies also showed that a physician’s communication style significantly impacts SDM in treating osteoarthritis [14]. Moreover, a physician’s confidence in medical interviews is positively correlated with SDM in primary care settings [15]. One study investigated the personality traits of medical residents related to the self-reported SDM and found that openness was significantly and inversely associated with SDM [16]. Thus, some physicians’ personality traits may affect SDM between physicians and patients through communication. However, no studies have investigated how physician personality traits are related to patient-rated SDM.

This study aimed to evaluate the association between attending physicians’ personality traits and SDM rated by their patients with SLE.

## Methods

### Study design and setting

Our cross-sectional study used baseline information from the Trust Measurement for Physicians and Patients with SLE (TRUMP2-SLE) multidisciplinary cohort study. The TRUMP2-SLE, conducted in 2020, enrolled patients from five academic medical centres. Data were obtained from electronic medical records and self-administered questionnaires completed by enrolled patients and their attending rheumatologists between June 2020 and August 2021. This study followed the tenets of the Declaration of Helsinki and the ethical guidelines for epidemiologic research in Japan. This study was approved by the ethics committee of Kochi Medical School (2024–69). All patients provided written informed consent to participate in the registry and for us to publish the study results.

### Study participants

The TRUMP2-SLE inclusion criteria were as follows: (1) age ≥20 years and diagnosed with SLE according to the revised 1997 American College of Rheumatology classification criteria [17]; (2) having received rheumatology care at one of the participating institutions and (3) ability to complete the questionnaire. All the treating rheumatologists were Japanese. Rheumatologists participating in the TRUMP2-SLE project were recruited from the participating institutions and voluntarily completed the self-reported questionnaires. The attending rheumatologists were identified based on the patients’ questionnaire responses.

### Outcome measures

The primary outcome was SDM, measured using the nine-item Shared Decision-Making Questionnaire (SDM-Q-9; Japanese version) [18, 19], a tool designed to assess the degree of patient involvement in the SDM process. The SDM-Q-9 consists of nine questions, each of which has a six-point scale of ‘completely disagree’ (0 points) to ‘completely agree’ (5 points) (Supplementary Data S1, available at *Rheumatology* online). The sum of the scores was converted into a scale of 0–100. Cronbach’s alpha coefficient for the Japanese version of the SDM-Q-9 was 0.917, indicating strong internal consistency [20]. The patients could complete the questionnaire in the waiting room or at home. The questionnaire text assured the participants that their responses would remain confidential, not be accessible to the attending rheumatologist, and be used solely for analysis at the central facility.

### Exposures

As the main exposure, we measured the Big Five personality traits of the attending rheumatologists using the Japanese version of the 10-Item Personality Inventory (TIPI-J) scale [21, 22]. The TIPI-J scale consists of 10 items (two for each domain) and is scored on a 7-point Likert scale (Supplementary Data S2, available at *Rheumatology* online). The five domains comprise extraversion, agreeableness, conscientiousness, neuroticism and openness [9]. A high extraversion level reflects sociability and emotional expressiveness, while a low extraversion level suggests introversion and shyness. Similarly, a high agreeableness level denotes kindness and altruism, whereas a low agreeableness level is associated with argumentativeness and coldness. A high conscientiousness level signifies orderliness and dutifulness, while a low conscientiousness level reflects carelessness and disorganization. A high level of neuroticism is characterized by significant anxiety and moodiness, while a low level of neuroticism indicates emotional stability and calmness. Finally, a high openness level denotes a broad range of interest and imagination, while a low openness level suggests conventionality and a narrow range of interests [23].

The attending rheumatologists were instructed to score each item on a scale of 1–7, with 1 signifying ‘disagree strongly’ and 7 corresponding to ‘agree strongly’. After reversing the scores for negatively worded items (Questions 2, 4, 6, 8 and 10 in Supplementary Data S2, available at *Rheumatology* online), each domain’s score was calculated by summing the item scores and then averaging the total [18, 24]. The rheumatologists understood the purpose of the project and completed the questionnaires outside of the clinical setting. We used the clusters derived from the TIPI-J scores, described later in the methods section, as the secondary exposure.

### Measurement of covariates

The included covariates were each attending rheumatologist’s age, sex, job title and number of SLE patients managed up to the survey. These covariates were selected because they could be associated with TIPI-J and SDM-Q-9 scores. The job titles were divided into associate professor or higher and lecturer or lower. We determined that patient variables did not affect the attending rheumatologist’s TIPI-J scores and, thus, were not confounding.

The attending rheumatologists determined the Systemic Lupus Erythematosus Disease Activity Index 2000 (SLEDAI-2K) and Systemic Lupus International Collaborating Clinic Damage Index (SLICC-DI) scores. Corticosteroid doses were recorded as prednisolone equivalents. Immunosuppressants were considered present if any of the following were prescribed: mycophenolate mofetil, azathioprine, tacrolimus, ciclosporin and other immunosuppressants.

### Statistical analysis

The descriptive statistics of the enrolled patients and attending rheumatologists are presented as the median and interquartile range (IQR) for continuous variables and as count (%) for categorical variables. Subsequently, the Mann–Whitney *U* test was used to compare the relationship between the SDM-Q-9 score and each of the five TIPI-J scores divided into two groups by the third quartile. Since we were particularly interested in the relationship between high TIPI-J scores and SDM, we chose the third quartile of the physicians’ TIPI-J scores as the cut-off value for dividing the groups.

Considering the clustering of SDM-Q-9 due to multiple patient ratings for the same attending rheumatologist, the associations between the five personality traits and SDM-Q-9 scores were analysed using linear mixed-effects models with cluster-robust variance estimation [25]. The four rheumatologist characteristics described above were included in the multivariate analysis as covariates. We also performed a similar analysis stratified by job title (job_1 [lecturer or lower] and job_2 [associate professor or higher]). As an exploratory analysis, we also performed a multivariate analysis that included patients’ age, sex, SLEDAI-2K, SLICC-DI, current immunosuppressant use as covariates and physician-related factors. Missing values were imputed using the multiple imputation method.

Additionally, a cluster analysis was performed using the TIPI-J scores of the 43 rheumatologists to detect relatively homogeneous groups with patterns of personality traits. Hierarchical clustering using Ward’s method was performed considering standardized scores and squared Euclidean distances. To decide the optimal number of clusters, a dendrogram was plotted and cut where the heights of the vertical lines were the longest [26]. Cluster consistency was assessed using Steel–Dwass tests. To validate the clustering results, we also performed a sensitivity analysis using the DIANA (DIvisive ANAlysis) clustering method.

Finally, the association between the identified clusters and the SDM-Q-9 scores was also analysed using a linear mixed-effects model with cluster-robust variance estimation. Statistical significance was set at *P* < 0.05. We conducted statistical analyses using Stata version 18.0 (StataCorp, College Station, TX, USA) and R version 4.4.1 (R Foundation for Statistical Computing, Vienna, Austria).

### Patients and public involvement

Neither the general public nor patients with SLE were involved in the study planning, recruitment or conduct processes.

## Results

### Patient and attending rheumatologist characteristics

Patient characteristics are present in Table 1. The median (IQR) age of the 493 enrolled patients was 45 (36–54) years; of them, 437 (88.6%) were women. The median (IQR) duration from disease onset was 10 (4–17) years. The median (IQR) duration with their attending rheumatologists was 5 (4–5) years. The median (IQR) SLEDAI-2K and SLICC-DI scores at the time of registration were 4 (2–6) and 0 (0–1), respectively. The median (IQR) SDM-Q-9 score was 75.6 (62.2–88.9).

**Table 1.** Patient characteristics (*N* = 493)

		Missing data, n
Demographics		
Age, median (IQR), years	45 (36–54)	0
Female patients, *n* (%)	437 (88.6)	0
Job title of attending physician		
Lecturer or lower, *n* (%)	280 (56.8)	0
Associate professor or higher, *n* (%)	213 (43.2)	0
Disease duration, median (IQR), years	10 (4–17)	7
Duration with their rheumatologist, median (IQR), years	5 (4–5)	8
SLEDAI-2K, median (IQR), pts	4 (2–6)	0
SLICC-DI, median (IQR), pts	0 (0–1)	5
History of relapses, *n* (%)	66 (14.5)	38
PSL, dose, median (IQR), mg/day	5 (2.5–7.0)	3
Current immunosuppressant use, *n* (%)	358 (73.1)	3
Current HCQ use, *n* (%)	233 (47.6)	0

HCQ, hydroxychloroquine; IQR, interquartile range; PSL, prednisolone; SLEDAI-2K, Systemic Lupus Erythematosus Disease Activity Index 2000; SLICC-DI, Systemic Lupus International Collaborating Clinics Damage Index.

The attending rheumatologists’ characteristics are present in Table 2. A total of 43 rheumatologists were enrolled. The median (IQR) age of the attending rheumatologists was 39 (34–43) years; 10 (23.3%) of them were women. Regarding job titles, 36 (83.7%) were at the level of lecturer or lower and seven (16.3%) were associate professor or higher. The number of SLE patients treated per rheumatologist up to the present survey was <10 for three (7.0%), 10–49 for 21 (48.8%), 50–99 for 11 (25.6%) and ≥100 for eight (18.6%). The number of patients per rheumatologist in charge in the present survey was <10 for 29 (67.4%), 11–20 for 10 (23.3%) and 21–94 for four (9.3%). The median (IQR) TIPI-J scores were 4.0 (3.0–5.0) for extraversion, 5.0 (4.5–5.5) for agreeableness, 3.0 (3.0–4.0) for conscientiousness, 3.5 (3.0–4.5) for neuroticism and 4.0 (3.0–5.0) for openness (Table 2).

**Table 2.** Attending rheumatologist characteristics and TIPI-J scores (*N* = 43)

Characteristics	
Demographics	
Age, median (IQR), years	39 (34–43)
Female rheumatologists, *n* (%)	10 (23.3)
Job title	
Lecturer or lower, *n* (%)	36 (83.7)
Associate professor or higher, *n* (%)	7 (16.3)
Number of patients served up to this survey, *n* (%)	
<10	3 (7.0)
10–49	21 (48.8)
50–99	11 (25.6)
100 ≤	8 (18.6)
Number of patients in charge, *n* (%)	
≤10	29 (67.4)
11–20	10 (23.3)
21–94	4 (9.3)

TIPI-J scores	Median (IQR)

Extraversion	4.0 (3.0–5.0)
Agreeableness	5.0 (4.5–5.5)
Conscientiousness	3.0 (3.0–4.0)
Neuroticism	3.5 (3.0–4.5)
Openness	4.0 (3.0–5.0)

No missing data.

IQR, interquartile range; TIPI-J, The Japanese version of the 10-Item Personality Inventory.

### Relationship between TIPI-J scores and SDM-Q-9 scores

**Figure 1.**
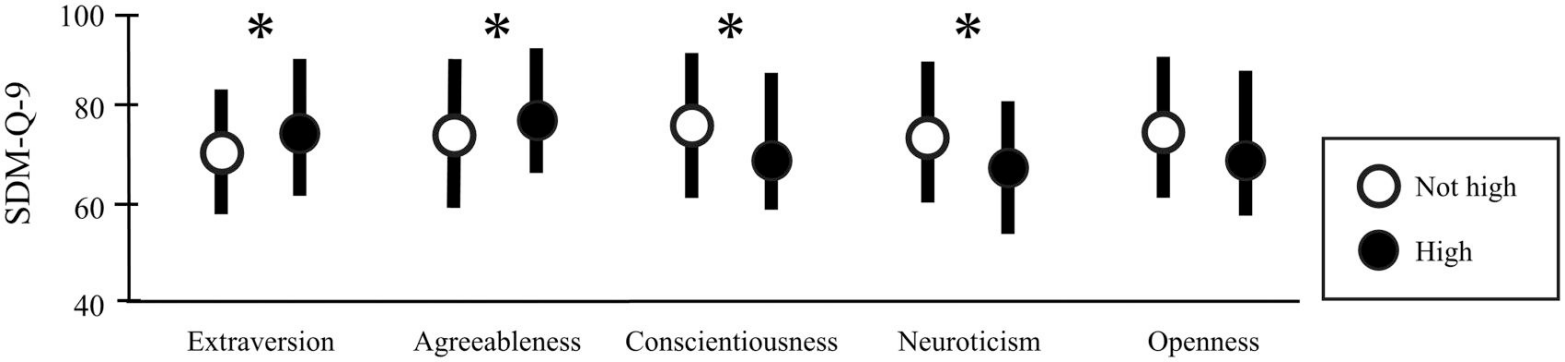
Association between SDM-Q-9 scores and rheumatologists’ personality traits, classified as high (>3rd quartile) or low (≤3rd quartile) based on TIPI-J scores. Error bars indicate interquartile ranges. IQR, interquartile range; SDM-Q-9, the 9-item Shared Decision Making Questionnaire; TIPI-J, the Japanese version of the 10-item Personality Inventory. *P < 0.05 (Mann–Whitney *U* test)

We divided the five TIPI-J scores into two groups—‘high’ and ‘not high’—according to the third quartile. High extraversion (score >5.0) and high agreeableness (score >5.5) levels were related to higher patients’ SDM-9 scores (median [IQR] of SDM-9 scores: 77.8 [62.2–91.1] with high extraversion level vs 75.6 [60.0–84.4] with not high extraversion level, *P* < 0.01; and 80 [67.8–93.3] for high agreeableness level vs 75.6 [60.0–88.9] for not high agreeableness level, *P* = 0.04, respectively). While high conscientiousness (>4.0) and high neuroticism (>4.5) levels were related to lower SDM-9 scores (median [IQR] of SDM-9 scores: 73.3 [60.0–86.7] for high conscientiousness level vs 77.8 [62.2–91.1] for not high conscientiousness level, *P* = 0.02; and 71.1 [57.8–82.2] for high neuroticism level vs 77.8 [62.2–91.1] for not high neuroticism level, *P* < 0.01, respectively). Openness level did not differ significantly in terms of SDM-9 scores (median [IQR] of SDM-9 scores: 73.3 [60–88.9] for high openness [>5.0] level vs 77.8 [62.2–91.1] for not high openness level, *P* = 0.46) (Fig. 1).

The linear mixed-effects model showed negative relationships between conscientiousness and SDM-Q-9 score and between neuroticism and SDM-Q-9 score even after the adjustment for confounding variables (Table 3). Stratified analysis by job title revealed similar patterns in the job_2 group (associate professor or higher), but not in the job_1 group (lecturer or lower) (Supplementary Table S1, available at *Rheumatology* online). Exploratory analysis, including patient-related factors, showed that the beta coefficients remained unchanged (Supplementary Table S2, available at *Rheumatology* online).

**Table 3.** Relationship between attending rheumatologists’ Big Five personal traits and SDM-Q-9

	*β*	95% CI	*P*-value
Extraversion	1.06	−1.22 to 3.35	0.363
Agreeableness	2.74	−0.02 to 5.49	0.051
Conscientiousness	−1.67	−3.02 to −0.33	0.015*
Neuroticism	−2.06	−4.08 to −0.04	0.045*
Openness	−0.84	−2.94 to 1.25	0.430

Linear mixed-effects models were employed using cluster-robust variance estimation, with each rheumatologist as a cluster unit. Control variables: (attending rheumatologists) age, sex, job title and number of SLE patients served up to this survey.

*β*, regression coefficient; SDM-Q-9, The 9-item Shared Decision Making Questionnaire.

* *P* < 0.05.

**Figure 2.**
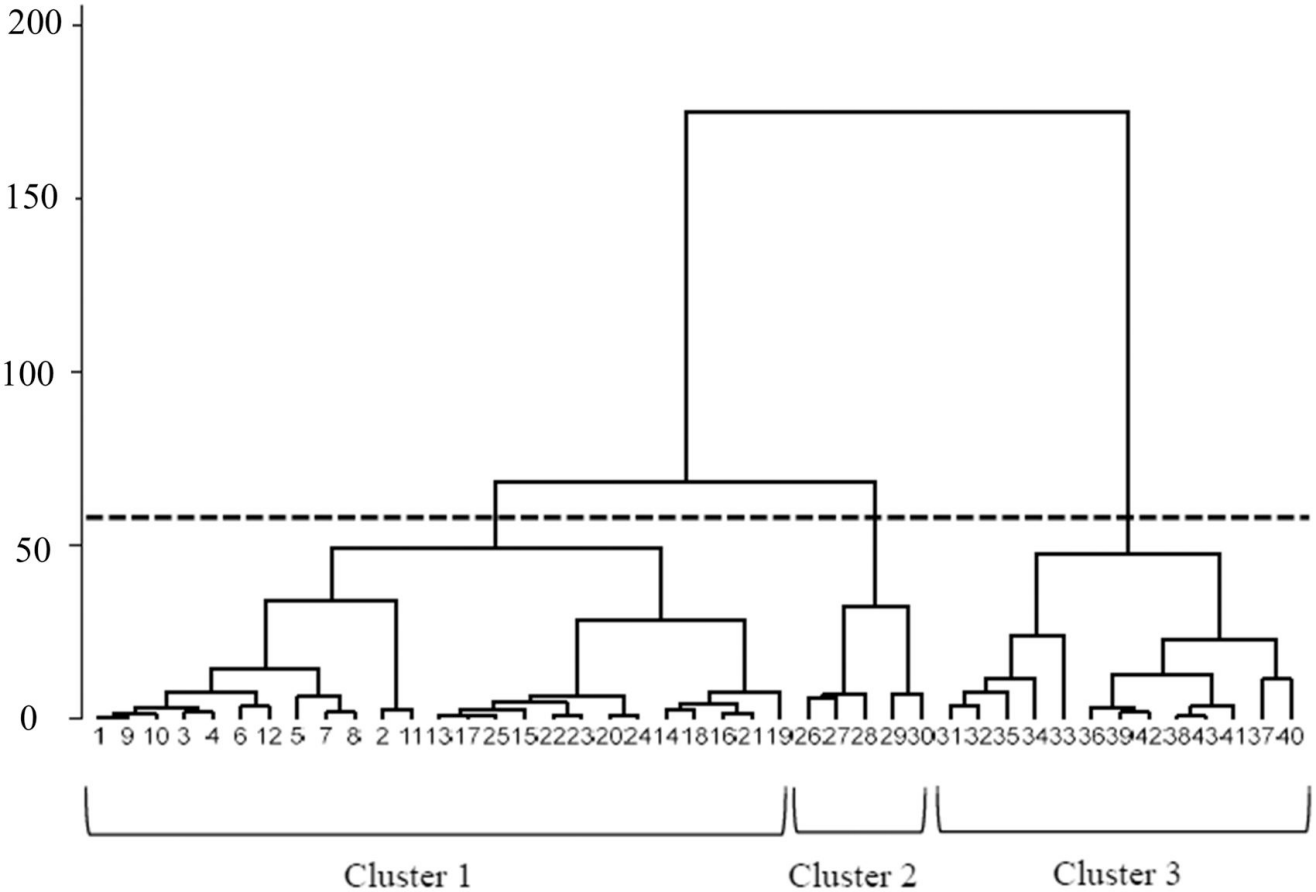
Dendrogram illustrating the cluster analysis of attending rheumatologists’ TIPI-J scores. Hierarchical cluster analysis (Ward’s method) based on TIPI-J scores of 43 rheumatologists. The dashed line indicates the cut-off level for the three clusters. TIPI-J, the Japanese version of the 10-Item Personality Inventory

A hierarchical cluster analysis of 43 rheumatologists produced a dendrogram that revealed three distinct clusters (Fig. 2). The comparison of scores for each TIPI-J component among the three clusters is shown in Supplementary Fig. S1, available at *Rheumatology* online. Cluster 1 (*n* = 25) was characterized by low extraversion and openness scores, Cluster 2 (*n* = 5) by low conscientiousness and neuroticism scores and Cluster 3 (*n* = 13) by high extraversion, neuroticism and openness scores. The extraversion scores were significantly lower in Cluster 1 than in Clusters 2 and 3. Cluster 2 had significantly lower conscientiousness scores than Cluster 3. For neuroticism, Cluster 2 had significantly lower scores than Clusters 1 and 3, while Cluster 3 scored significantly higher than Cluster 1. The openness scores were significantly higher in Cluster 3 than in Cluster 1. Sensitivity analysis using the DIANA clustering method revealed a very similar three-cluster structure (Supplementary Table S3, available at *Rheumatology* online).

Table 4 shows the relationship between the three clusters of attending rheumatologists and the SDM-Q-9 score of patients with SLE, analysed using a linear mixed-effects model. When Cluster 2 (*n* = 48) was used as the reference group, Clusters 1 (*n* = 221) and 3 (*n* = 224) were significantly correlated to lower SDM-Q-9 scores (regression coefficient [95% CI]: Cluster 1, −8.23 [−16.10 to −0.36]; and Cluster 3, −8.23 [−15.23 to −1.24]).

**Table 4.** Relationship between the three clusters of attending rheumatologists and SDM-Q-9

	*N*	*β*	95% CI	*P*-value
Cluster 1	221	−8.23	−16.10 to −0.36	0.040*
Cluster 2	48	Reference		
Cluster 3	224	−8.23	−15.23 to −1.24	0.021*

A linear mixed-effects model was employed using cluster-robust variance estimation, with each rheumatologist as a cluster unit.

Control variables: (attending rheumatologists) age, sex, job title and number of SLE patients served up to this survey.

*β*, regression coefficient; SDM-Q-9, The 9-item Shared Decision Making Questionnaire.

^*^ *P* < 0.05.

## Discussion

To our knowledge, this is the first study to examine how physician personality traits relate to patient-rated SDM. Previous studies focused on residents’ personality traits and self-rated SDM skills [16]. Conscientiousness and neuroticism were negatively associated with SDM. A physician cluster characterized by low conscientiousness and neuroticism demonstrated better SDM compared with the other two clusters.

Physicians’ conscientiousness in pursuing treatment goals may have a negative impact on the patient relationship in the context of SDM. Several previous studies investigated the impact of the Big Five personality traits on medical practice and communication styles. Physicians generally exhibit higher neuroticism levels than the general population [25, 27], which increases vulnerability to stress, burnout and emotional exhaustion—factors that may compromise empathy and impair patient involvement in decision-making [27]. Although conscientious physicians are often motivated and accurate diagnosticians [28], they are less likely to elicit patients’ preferences [29] and be more inclined to recommend aggressive treatments, particularly for vulnerable populations for elderly leukaemia patients [30]. Our previous study also confirmed that lower patient trust levels were associated with higher physician conscientiousness scores [25]. High conscientiousness is characterized by diligence, a sense of responsibility and planning, and is generally considered a positive trait. However, excessive self-control and a goal-oriented attitude can negatively impact interpersonal relationships [31]. The associations between personality traits and SDM were stronger among physicians with higher job titles compared with those with lower titles. Generally, higher job titles entail greater responsibility, which may lead physicians towards more paternalistic decision-making [32]. Such increased paternalistic tendencies might amplify the negative association of physicians’ personality traits on SDM in the higher job-title group.

Perfectionism, closely related to both conscientiousness and neuroticism, may explain some of these effects. It involves setting excessively high standards and strict self-evaluations [33]. Perfectionism has two aspects: concern, characterizes by fear of mistakes and self-doubt and striving [14], involving high personal expectations and a belief that perfection is essential [34]. Several studies have shown that perfectionist concern is strongly correlated with neuroticism, while perfectionist striving is moderately correlated with conscientiousness [35–37]. The anxiety and stress accompanying these traits may underlie the lower SDM scores seen in our study.

People with high conscientiousness levels may unconsciously expect others to meet similar standards, making collaborative communication difficult. Organizational psychology research has shown that high conscientiousness levels can lead to friction and frustration among team members [38] and inflexibility in interpersonal relationships, reducing empathy [39] and potentially leading to patient dissatisfaction [40]. Our findings suggest that such tendencies may also undermine SDM. Indeed, the relationship between conscientiousness and job performance may follow an inverse U-shape curve [41] in which moderate conscientiousness is associated with the most desirable outcomes, while extremely high or low conscientiousness levels may result in poor performance. Our study findings suggest that physicians should avoid excessive perfectionism and instead focus on patient dialogue and flexibility, which could improve SDM.

Our study identified three clusters of physicians by their TIPI-J scores. One cluster, characterized by low conscientiousness and low neuroticism, was significantly associated with higher patient-rated SDM. A cluster analysis based on employee personality traits in the business sector found that high conscientiousness and high neuroticism clusters were associated with job performance, whereas excessive demands for perfection increased mental strain and decreased job satisfaction [42]. Unlike that study, we did not identify any clusters with high levels of both traits. While we were able to identify clusters associated with good SDM, we could not determine specific populations where SDM interventions would be most needed.

Our study findings may be interpreted through the concept of a professional ‘persona’ that doctors develop when interacting with patients. A professional persona is formed by controlling one’s emotions and adhering to professional norms, thereby allowing physicians to remain calm and objective. Highly neurotic physicians may struggle to maintain this persona and allow their emotional instability to negatively impact patient communication, leading patients to perceive them as uncooperative and rate their SDM lower [43]. Conscientiousness is typically associated with attentiveness and responsibility, but excessively high conscientiousness levels can lead to controlling, rigid behaviours and perfectionism to the point that physicians dominate conversations and downplay their patients’ opinions. In contrast, physicians with low conscientiousness levels tend to take a more flexible approach, making it easier for patients to express their views and participate in SDM processes [44]. The concept of professional personas is well described in medical education literature [45, 46]. Future research should explore how physicians balance their inherent personality traits and professional personas, especially in SDM situations. This could improve medical education and training programs aimed at enhancing SDM skills. Physicians with strong self-monitoring skills—those who can control their behaviours and emotions—have adjusted their communication style to meet their patients’ needs, leading to better care quality [47].

This study has several limitations. First, the Big Five personality traits of physicians were assessed via self-reported questionnaires, which are subjective. The positive correlations were reported for extraversion, conscientiousness and openness, whereas no correlation was found for agreeableness and neuroticism between self- and externally-rated assessments [22]. It is possible that the self-assessment of TIPI includes social desirability bias. However, this may also indicate that physicians’ self-assessments reflect a professional persona rather than an inherent personality. We speculate that the physicians’ self-assessment using TIPI-J may be modifiable through education or training. Second, although this was a multicentre study, it included only Japanese academic centres; thus, rheumatologists and patients with SLE may differ from those in other clinics and general hospitals in Japan. Third, it is unclear whether these results can be generalized to countries with different cultural and social backgrounds; thus, further research is needed to validate our findings.

In conclusion, this study found that higher conscientiousness and neuroticism levels among rheumatologists were associated with lower SDM-Q-9 scores according to their patients with SLE. A physician’s perfectionist tendencies may be a barrier to the SDM process with patients. Further research is warranted to determine whether educational interventions that shape professional personas can enhance SDM.

## Supplementary Material

keaf288_Supplementary_Data

## Data Availability

The datasets generated and analysed during the study are available from the corresponding author upon reasonable request.
