# Supplementary material for: Association of physicians’ Big Five personality traits with shared decision-making in patients with SLE
Source: Rheumatology (Oxford). 2025 May 26;64(10):5269–76. doi: 10.1093/rheumatology/keaf288 (PMC12494213; doi:10.1093/rheumatology/keaf288)
Supplement: keaf288_Supplementary_Data [file keaf288_supplementary_data.zip › keaf288_Supplementary_Data/rhe-25-0221-File005.docx]

**Supplementary Data S1. Ten-Item Personality Inventory (TIPI)**

Here are a number of personality traits that may or may not apply to you. Please write a number next to each statement to indicate the extent to which you agree or disagree with that statement. You should rate the extent to which the pair of traits applies to you, even if one characteristic applies more strongly than the other.

1= Disagree strongly, 2= Disagree moderately, 3= Disagree a little,
4= Neither agree nor disagree, 5= Agree a little, 6= Agree moderately, 7= Agree strongly

I see myself as:

1. ___Extraverted, enthusiastic.
2. ___Critical, quarrelsome.
3. ___Dependable, self-disciplined.
4. ___Anxious, easily upset.
5. ___Open to new experiences, complex.
6. ___Reserved, quiet.
7. ___Sympathetic, warm.
8. ___Disorganized, careless
9. ___Calm, emotionally stable.
10. ___Conventional, uncreative.

The scoring for the original version (TIPI) can be calculated according to the following formula.

1. Extraversion = ( Question 1 + [8 － {Question 6}] ) / 2

2. Agreeableness = ( Question 7 + [8 － {Question 2}]) / 2

3. Conscientiousness = ( Question 3 + [8 － {Question 8}] ) / 2

4. Neuroticism = ( Question 9 + [8 － {Question 4}]) / 2

5. Openness = ( Question 5 + [8 － {Question 10}]) / 2

**Supplementary Data S2. The 9-item Shared Decision Making Questionnaire (SDM-Q-9)**

Nine statements related to the decision-making in your consultation are listed below. For each statement, please indicate how much you agree or disagree. 1 = completely disagree, 2 = strongly disagree, 3 = somewhat disagree,
4 = somewhat agree, 5 = strongly agree, 6 = completely agree

1. ___ My doctor made clear that a decision needs to be made.

2. ___My doctor wanted to know exactly how I wanted to be involved in making the
 decision.

3. ___My doctor told me that there are different options for treating my medical condition.

4. ___My doctor precisely explained the advantages and disadvantages of the treatment
options.

5. ___My doctor helped me understand all the information.

6. ___My doctor asked me which treatment option I prefer.

7. ___My doctor and I thoroughly weighed the different treatment options.

8. ___My doctor and I selected a treatment option together.

9 ____My doctor and I reached an agreement on how to proceed.

The scoring for SDM-Q-9

1. The SDM-Q-9 consists of nine questions, each of which patients answer using a 6-point scale.
2. Patients were asked to choose one of the following responses for each item: ‘completely disagree’ (0 points) to ‘completely agree’ (5 points).
3. The sum of the scores was converted into a scale ranging from 0 to 100.

**Supplementary Table S1. Relationship between attending rheumatologists’ Big Five personal traits and SDM-Q-9: Job title stratification analysis**

|  | β | 95% CI | *p*-value |
| --- | --- | --- | --- |
| Extraversion |  |  |  |
| Overall | 1.06 | -1.22 to 3.35 | 0.363 |
| job_1 | 0.81 | -1.08 to 2.70 | 0.400 |
| job_2 | -9.16 | -54.39 to 36.06 | 0.691 |
| Agreeableness |  |  |  |
| Overall | 2.74 | -0.02 to 5.49 | 0.051 |
| job_1 | 1.12 | -0.97 to 3.21 | 0.294 |
| job_2 | 19.70 | -10.47 to 28.93 | <0.01** |
| Conscientiousness |  |  |  |
| Overall | -1.67 | -3.02 to -0.33 | 0.015 * |
| job_1 | 0.98 | -0.28 to 3.23 | 0.126 |
| job_2 | -5.62 | -7.87 to -3.37 | <0.01** |
| Neuroticism |  |  |  |
| Overall | -2.06 | -4.08 to -0.04 | 0.045* |
| job_1 | 0.73 | -1.24 to 2.09 | 0.468 |
| job_2 | -4.13 | -5.76 to -2.69 | <0.01** |
| Openness |  |  |  |
| Overall | -0.84 | -2.94 to 1.25 | 0.430 |
| job_1 | -1.08 | -3.11 to 0.94 | 0.294 |
| job_2 | -10.02 | -23.24 to 3.19 | 0.137 |

Linear mixed-effects models were employed, utilizing cluster-robust variance estimation, with each rheumatologist serving as the cluster unit.

Control variables: (attending rheumatologists) age, sex and the number of SLE patients served up to the time of this survey.

β, regression coefficient; CI, confidence interval; job_1, lecturer or lower; job_2, associate professor or higher; SDM-Q-9, The 9-item Shared Decision Making Questionnaire. **p* < 0.05, ***p* < 0.01

**Supplementary Table S2. Relationship between attending rheumatologists’ Big Five personal traits and SDM-Q-9 (adjusted for patient covariates)**

|  | β | 95% CI | *p*-value |
| --- | --- | --- | --- |
| Extraversion | 1.54 | -0.76 to 3.83 | 0.190 |
| Agreeableness | 2.74 | 0.03 to 5.44 | 0.048* |
| Conscientiousness | -1.69 | -3.01 to -0.36 | 0.013* |
| Neuroticism | -2.21 | -4.24 to -0.18 | 0.033* |
| Openness | -0.70 | -2.80 to 1.41 | 0.516 |

Linear mixed-effects models were employed, utilizing cluster-robust variance estimation, with each rheumatologist serving as the cluster unit.

Control variables: (attending rheumatologists) age, sex, job title, and number of SLE patients served up to this survey. (SLE patients) age, sex, SLEDAI-2K, SLICC-DI, and current immunosuppressant use.

β, regression coefficient; CI, confidence interval; HCQ, hydroxychloroquine; SDM-Q-9, The 9-item Shared Decision Making Questionnaire; SLEDAI-2K, Systemic Lupus Erythematosus Disease Activity Index 2000; SLICC-DI, Systemic Lupus International Collaborating Clinics Damage Index. *p < 0.05

**Supplementary Table S3. Comparison of cluster analysis results using Ward’s method and the Divisive Analysis method**

|  | Ward’s method | | |  | DIANA method | | |
| --- | --- | --- | --- | --- | --- | --- | --- |
|  | Cluster 1  n = 25 | Cluster 2  n = 5 | Cluster 3  n = 13 |  | Cluster 1  n = 26 | Cluster 2  n = 4 | Cluster 3  n = 13 |
| Extraversion | 3.24 (1.01) | 4.90 (1.24) | 5.27 (1.07) |  | 3.31 (0.96) | 3.50(1.00) | 5.69 (0.78) |
| Agreeableness | 5.04 (0.58) | 3.60 (1.47) | 4.81 (0.99) |  | 5.06 (0.57) | 3.38 (1.25) | 4.73 (1.09) |
| Conscientiousness | 3.30 (0.91) | 2.40 (0.89) | 4.27 (1.48) |  | 3.46 (0.97) | 2.00 (1.00) | 4.00 (1.46) |
| Neuroticism | 3.52 (0.81) | 2.40 (0.55) | 5.15 (1.03) |  | 3.71 (0.83) | 2.12 (0.75) | 4.77 (1.38) |
| Openness | 3.36 (0.98) | 4.50 (1.27) | 4.96 (0.90) |  | 3.44 (0.96) | 3.38 (0.85) | 5.23 (0.83) |

Values are shown as mean (standard deviation).

DIANA, Divisive Analysis.
