# Supplementary figures and images for: Association of physicians’ Big Five personality traits with shared decision-making in patients with SLE
Source: Rheumatology (Oxford). 2025 May 26;64(10):5269–76. doi: 10.1093/rheumatology/keaf288 (PMC12494213; doi:10.1093/rheumatology/keaf288)

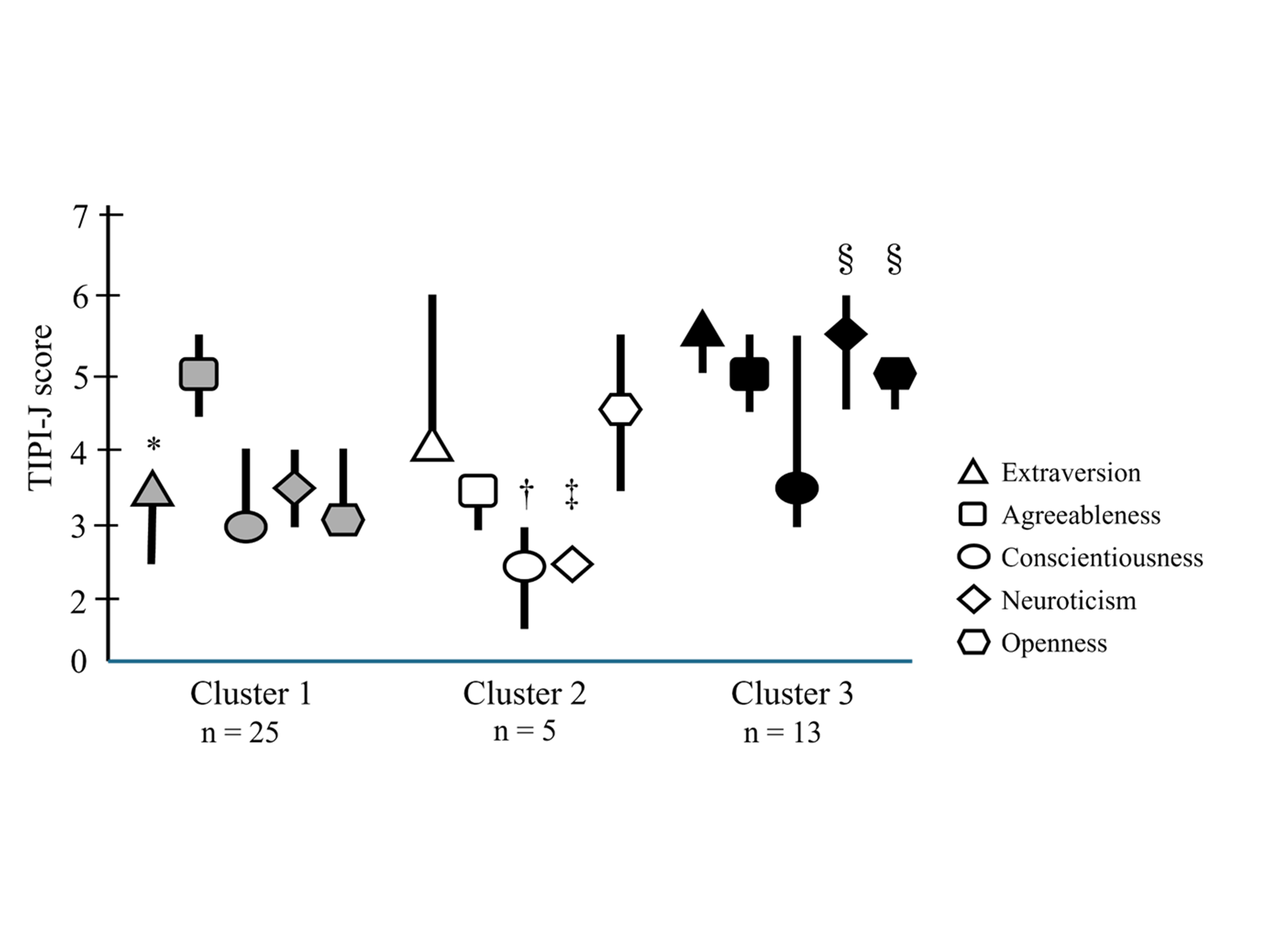

Supplement: keaf288_Supplementary_Data [file keaf288_supplementary_data.zip › keaf288_Supplementary_Data/rhe-25-0221-File006.TIF]
